# Melamine promotes calcium crystal formation in three-dimensional microfluidic device

**DOI:** 10.1038/s41598-018-37191-5

**Published:** 2019-01-29

**Authors:** Farai Gombedza, Sade Evans, Samuel Shin, Eugenia Awuah Boadi, Qian Zhang, Zhihong Nie, Bidhan C. Bandyopadhyay

**Affiliations:** 1 Calcium Signaling Laboratory, Research Service, Veterans Affairs Medical Center, 50 Irving Street, NW, Washington, DC 20422 USA; 2 Department of Chemistry and Biochemistry, University of Maryland, College Park, MD 20742 USA

## Abstract

Melamine, which induces proximal tubular (PT) cell damage has a greater nephrotoxic effect when combined with cyanuric and uric acids; however, it is unknown whether such effect can stimulate calcium phosphate (CaP)/calcium oxalate (CaOx) stone formation. Here, we show that melamine acts as an inducer of CaP, CaOx and CaP + CaOx (mixed) crystal formations in a time and concentration-dependent manner by stabilizing those crystals and further co-aggregating with melamine. To explore the physiological relevance of such melamine-augmented calcium crystal formation, we used 2-dimensional (2D) and 3D microfluidic (MF) device, embedded with PT cells, which also resembled the effect of melamine-stimulated CaP, CaOx and mixed crystal formation. Significantly, addition of preformed CaP and/or CaOx crystal in the presence of melamine, further potentiated those crystal formations in 3D MFs, which helped the growth and aggregation of mixed crystals. Our data show that the mechanism of such predisposition of stone formation could be largely due to co-crystallization between melamine and CaP/CaOx and pronounced effect on induction of stone-forming pathway activation in 3D MF. Taken together, melamine-induced CaP and/or CaOx crystal formation *ex-vivo* will help us in understanding the larger role of melamine as an environmental toxicant in producing the pathology in similar cellular microenvironments.

## Introduction

The prevalence and frequency of calcium stones, which account for more than 70% of all nephrolithiasis, increased in the last decade^1,2^. A majority of these renal stones consist of calcium oxalate (CaOx), however, they are often mixed with variable amounts of calcium phosphate (CaP)^3^. Although the mechanism of aggregation of these CaP crystals (amorphous) is unknown, such crystals are proposed to act as a nidus for CaP + CaOx (mixed) crystal^4–7^. Biomineralization of these mixed crystals result in inflammation and cell injury, expanded by the calcification of collagen and membranous vesicles^8,9^, and eventually leads to the formation of CaOx stones^10^. Although extensive research on stone formation has been conducted over the past century, the mechanism by which crystals nucleate, grow, and aggregate into stones is still poorly understood. In order to improve our understanding of the origin and the mechanism of such biologically controlled mineralization, we have developed a novel MF-based workbench that can simulate the dynamical biological conditions of an *in vivo* renal tubular system^11^. A recently described publication attempted to show CaOx crystals in micro-calcification using MF-based system^12^, however, such a finding lacks the essential cellular microenvironment as a biological component to make it relevant to the pathological and physiological comparisons in CaP/CaOx crystal formation *in situ*.

Over the past several years there has been a worldwide upsurge in the prevalence and frequency of kidney stone diseases^13^. Among the possible factors for this increase, a synthetic compound, melamine, which has a broad spectrum use in plastics, dishware, kitchen utensils and adhesives^14^, has been shown to contribute to acute kidney injury and kidney stone diseases^15^. Several studies along these lines identified the downstream intracellular pathways of melamine-induced renal cytotoxicity resulting in increased inflammation and oxidative stress^16^ and excessive intracellular reactive oxygen species (ROS) production^17^, leading to apoptosis. In addition, our recent findings show that the mechanism of action of melamine in proximal tubular (PT) cell death is via the extracellular Ca^2+^-sensing receptor-induced Ca^2+^ signaling pathway^18^. Several studies have indicated that PT cell death may contribute to CaP or CaOx stone formation^19^. More importantly, our previous findings show that melamine induced a sustained intracellular [Ca^2+^] ([Ca^2+^]_i_) response, a component which can cause a disturbance in apico-basal [Ca^2+^]_i_ mobilization required for transepithelial Ca^2+^ transport^20^. Indeed, such a compromise in Ca^2+^ clearance due to the exposure of melamine in contaminated food could have an implication on non-cyanurate stone formation such as CaP/CaOx stones^21^. Moreover, excess Ca^2+^ due to a compromise in transcellular Ca^2+^ transport can further contribute to CaP crystal formation in the loop of Henle (LOH)^22^, resulting in mixed stone formation^23^. Furthermore, resulting melamine exposure induced oxidative stress and cell death can carry over cell debris and lipids to the downstream segment, which are all factors that contribute to crystal growth in such non-cyanurate kidney stone formation^24^. Although some clinical studies indicate the predisposing scenario^25^, no study was found examining the effect of melamine in CaP/CaOx stone formation. We show here the contribution of melamine in CaP, CaOx and mixed stone formation, including a cellular microenvironment, and went above and beyond by utilizing a *vivo*-like 3D MF cellular environment to explore such effects of melamine exposure, ensuring the possibility and its pathophysiological relevance in CaP/CaOx stone formation.

## Methods

### Materials

Dulbecco’s Modified Eagle Medium (DMEM), Fetal Bovine Serum (FBS), penicillin and streptomycin were purchased from Invitrogen (Carlsbad, CA). Melamine was purchased from Sigma-Aldrich (St. Louis, MO) and a stock solution of 25 mM was prepared by dissolving melamine in ddH2O at room temperature (RT; 25 °C). Other chemicals were purchased from Sigma-Aldrich.

### Alizarin Red staining

Alizarin red (3,4-Dihydroxy-9, 10-dioxo-2-anthracenesulfonic acid sodium) staining was done as previously described to detect the presence of CaP and/or CaOx crystals^26,27^. CaP or CaOx solutions, cells cultured in 6 well plates, or MF devices were rinsed with Hank’s Buffered Salt Solution (HBSS) without phenol red, Ca^2+^ and Mg^2+^, fixed with 3% paraformaldehyde for 10 min at RT, then washed with HBSS 3 times. Crystals/cells were then incubated in 2% alizarin red solution (pH = 4.1 or 6.8) and incubated at 37 °C for 15 min. Stained images were obtained using Zeiss Axiovision microscope. Alizarin red stained images were quantified using ImageJ as previously described^28^.

### Extraction of Alizarin Red

To quantify CaP, CaOx and mixed crystal formation, alizarin red stains were extracted and quantified by spectrophotometric analysis as previously described^29^. Briefly, solutions were extracted by 10% acetic acid then incubated at RT with shaking for 30 min, placed in a hot water bath for 10 min at 85 °C. Afterwards samples were incubated for 5 min on ice, then centrifuged at 12,700 rpm (15,000 g) for 20 min. Following centrifugation, solution was added to 0.4 equivalence of 10% ammonium hydroxide. Samples were diluted 1:10 with HBSS and absorbance was read at 405 nm using Spectra Max^e^ microplate reader (Molecular Devices, San Jose, CA).

### Oil Red O staining

Oil red O staining was performed to detect the presence of melamine^30^. In accord with the design of alizarin red staining, solutions were collected and an equal volume of oil red O solutions was added. Samples were incubated at 37° for 72 hours. Stained images were obtained using Zeiss Axiovision microscope.

### CaP and CaOx synthesis

CaP saturated solution was prepared by mixing solutions of 2.4 mM calcium chloride (CaCl_2_), 0.9 mM disodium phosphate (Na_2_HPO_4_) and 5.8 mM monosodium phosphate (NaH_2_PO_4_). Preformed CaP crystals were prepared by mixing solutions of 2.9 mM CaCl_2_, 6.0 mM Na_2_HPO_4_ and 4.0 mM NaH_2_PO_4_. CaOx solution was prepared by mixing solutions of 2.4 mM calcium chloride (CaCl_2_), 1.0 mM sodium oxalate (Na_2_C_2_O_4_). Preformed CaOx crystals were prepared by mixing solutions of 2.4 mM CaCl_2_, 4.9 mM Na_2_C_2_O_4_. Solutions and crystals were prepared in HBSS. For crystals, mixed solutions were agitated for 30 min at RT and then centrifuged for 5 min (10,000 rpm), the supernatant was discarded and the crystals were washed twice with HBSS.

### Time-dependence in crystal formation and its dissolution

Preformed CaP, CaOx and mixed crystals were prepared as described above. Saturated solutions were exposed to with and without melamine (3 mM) for 0, 1, 3 and 10 minutes  (mins)  and measured the rate of crystal formations. Preformed crystals were incubated at RT^31^ with Millipore water with and without Melamine (3 mM) for 10 minutes to observe the crystal stability by measuring crystals remaining in solution^32^. Calcium crystals were then differentially stained with alizarin red (pH = 4.3 and 6.8) and quantified.

### Cell culture of human kidney cells and PT cells

The human kidney cell line (HK2), was purchased from Lonza (Walkersville, MD). HK2 cells were cultured in DMEM supplemented with 10% FBS, 2 mM glutamine, and 1% penicillin and streptomycin at 37 °C in 5% CO_2_. Primary PT cells were isolated from C57/BL6 mice as previously described^33^.

### Assembly of the MF device

MF devices were assembled as previously described^11^. To simulate an *in vivo* 3-D environment, polydimethylsiloxane (PDMS) microchannels were prepared by using a PDMS elastomer kit purchased from DOW Corning (Midland, Michigan) and disposable micro-sampling pipettes purchased from DOW Corning. PDMS pre-polymer and cross-linker were mixed to form a PDMS precursor. The mixture was then cured in an oven at 80 °C for 20 min and the Y-shaped channel was fabricated using three pieces of glass pipettes embedded into the PDMS block.

### MF Device Coating

MF device coating was carried out as previously described^11^. The MF device channels were rinsed with deionized H_2_O and then injected with tetraethyl orthosilicate solution and incubated at 120 °C to form a glass coating around the surface of the inner channel. The channels were then rinsed with water and sterilized under UV for 30 min. After sterilization, the channels were injected with fibronectin (1 mg/mL) and incubated for 3 hours at 37 °C to allow for adhesion of cells to the walls of the channel.

### Injection of cells into MF device

HK2 cells were seeded at 1 × 10^6^ cells/mL directly into the channel using a 25-gauge syringe pump. The device was placed in an incubator at 37 °C for 30 min to allow the cell to attach to the fibronectin coated MF channel surface. The device was turned over 180° and cells were reseeded to ensure that the surface of the MF channel was seeded with cells.

### Crystal induction in 2D cellular model

Cells were seeded and grown to 80% confluency in complete media. For crystal induction, appropriate crystal (CaP, CaOx or mixed crystals) (800 µg/ml) were added in serum-free media with or without melamine (0.1 mM, 0.3 mM or 1 mM) and incubated for 4 h at 37 °C in 5% CO_2_. After 4 h incubation, complete media was added and cells were incubated for a further 12–18 h.

### Crystal induction into the 3D cellular model

After seeding the cells, the MF devices were submerged with media inside Petri-dishes and kept in CO_2_ incubator for 5–6 hours for the cells to attach to the channels and conform the tubular structure. These devices were then used to examine the effect of melamine on crystal formation by continuously delivering media with a syringe pump to the luminal side via the inlet tubing with a flow rate of 0.1 mL/h to prevent cell detachment by shear force. The appropriate crystal conditions (CaP, CaOx, and mixed) with or without melamine were delivered into the device via another inlet. Strainer screen was used to measure the amount of crystals used for the MF device and were filtered to approximately 5–8 µM in size^34^.

### H_2_O_2_ release assay measurement

Release of H_2_O_2_ from cells in 3D MFs was assessed using H_2_O_2_ Cell-Based Assay Kit (Cayman). Briefly, cells within the MF devices were treated with different crystals conditions (CaP, CaOx, and mixed), with or without melamine. Pre-assay and assay preparations were done by following the instructions of the manufacturer. H_2_O_2_ release was assessed by subtracting the background wavelength (540 nm) from the emission wavelength (590 nm).

### Necrosis and apoptosis detection in MF devices through Annexin V/PI staining

After the HK2 cells were seeded into the MF devices, different crystal conditions (CaP, CaOx, and mixed) were adjusted into each of the MF devices. Melamine treatments were implemented through 30 minute pre-incubation in the device before the addition of crystals. Both apoptotic and necrotic cells were assessed by using Alexa Fluro 488 Annexin V/Dead Cell apoptosis Kit (Thermoscientific) as described previously^18^.

### Lactate dehydrogenase (LDH) assay

LDH release from crystal-treated HK2 cells in 3D MF was measured by using Pierce LDH Cytotoxicity Assay kit (Thermo Scientific). Following the treatment of crystals (Control, CaP, CaOx, mixed) within the HK2 cells, luminal fluids from MF device were extracted and immediately transferred to a 96 well plate in triplicates. LDH release reactions were done and the assessed by the subtraction of the absorbance at 680 nm (background) from the absorbance at 490 nm.

### RNA extraction and RT-PCR

Total RNA’s were isolated from cells cultured in MF devices using TRIzol as previously described^35^. Subsequently, DNAase treatments were performed and RNA concentrations were measured using nanodrop spectrophotometer. Afterwards, a cDNA synthesis kit (Promega Madison, WI) was used to reverse transcribe the RNA into the cDNA, which were amplified with gene specific primers (Supplementary Fig. 1A) purchased from Invitrogen and Integrated DNA Technologies (Coralville, IA) using the master mix PCR amplification reagent (Promega) as described^18^. With a T100 Thermocycler (Bio-Rad, Hercules, CA), following conditions for PCR have been used: one initial cycle at 95 °C for 3 min; 30–35 cycles of denaturation at 95 °C for 30 s, annealing at 55 °C for 30 s, and elongation at 72 °C for 45 s; an additional 5 min at 72 °C; and a final hold at 4 °C.

### Raman spectroscopy

To prepare Raman test, 2 μL of the sample was carefully dropped on a silicon wafer and dried. Raman measurements were performed by using a Horiba Jobin Yvon Aramis confocal Raman microscope (HORIBA Scientific, Edison, NJ) equipped with Laser Quantum MPC 6000 with a Nd: YAG laser (λ = 532 nm). Raman data was collected with a 10× objective lens, resulting in a spot size of 2.6 μm and a measured laser power of 5.44 mW on the sample. The grating was set as 600 gr/mm; the hole size was set as 400 μm with a total data sample accumulation time of 100 seconds.

### Statistical analysis

The results were reported as the mean ± SE and were analyzed using two-tailed t-test in Origin 6.1 software. The significant difference levels were set at *p < 0.05 and **p < 0.01. Details of all statistical analysis are mentioned in figures and figure legends.

## Result

### Identification of melamine, CaP and CaOx crystals by differential staining

**Figure 1:**
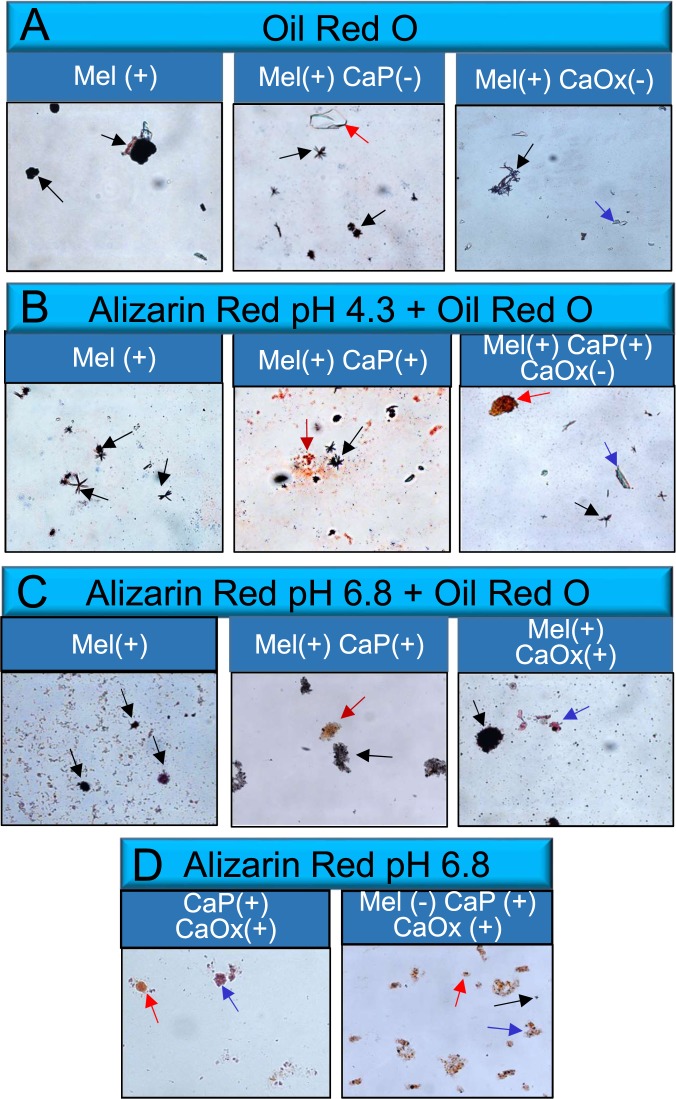
Crystal identification by differential staining. Melamine (Mel), calcium phosphate (CaP), and Calcium Oxalate (CaOx) crystal formations in non-cellular environment were stained with Oil Red O, alizarin red 4.3, and/or alizarin red 6.8 for differential staining, respectively (**A–D**). Black arrows point to Mel crystals, red arrows point to CaP crystals, and blue arrows point to CaOx crystals. Images at 20x magnification. Different methods of staining for Mel, CaP, and CaOx crystal formations: Positive Control (**+**); Negative Control (**−**)^26,27,30^.

The majority of kidney stones are composed of CaP, CaOx and mixed crystals. Melamine has previously been implicated in renal inflammation, oxidative stress and cytotoxicity^15^. Further, clinical studies have shown that melamine contributes to kidney stone formation^15^. To explore the effect of melamine on crystal formation, we added melamine alone as control, melamine to CaP solution, CaOx solution and mixed solutions. To confirm the identity of crystals formed, we differentially stained melamine, CaP and CaOx crystals using oil red O and alizarin red stain at pH 4.3 and 6.8 (Supplementary Fig. 1B). Oil Red O stained starlet-shaped melamine crystals but did not stain either CaP or CaOx crystals (Fig. 1A). Alizarin red stain (3,4-Dihydroxy-9, 10-dioxo-2-anthracenesulfonic acid sodium stain) a prominent red dye has been used to differentially stain CaP and CaOx crystals at pH 6.8 but not at pH 4.3^30^. Combinations of crystals; melamine alone, melamine and CaP, melamine and mixed crystals were stained with alizarin red 4.3 and oil red O stain (Fig. 1B). And melamine alone, melamine and CaP, melamine and CaOx, mixed alone, and melamine and mixed crystals were stained with alizarin red 6.8 and oil red O stain (Fig. 1C) and alizarin red 6.8 stain alone (Fig. 1D). We confirmed that in our lab, melamine was stained with oil red O stain, CaP and CaOx crystals were differentially stained with alizarin red stain at different pH conditions and utilized alizarin red stain for subsequent crystal staining experiments.

### Melamine promotes CaP and CaOx crystal formation in a non-cellular environment

**Figure 2:**
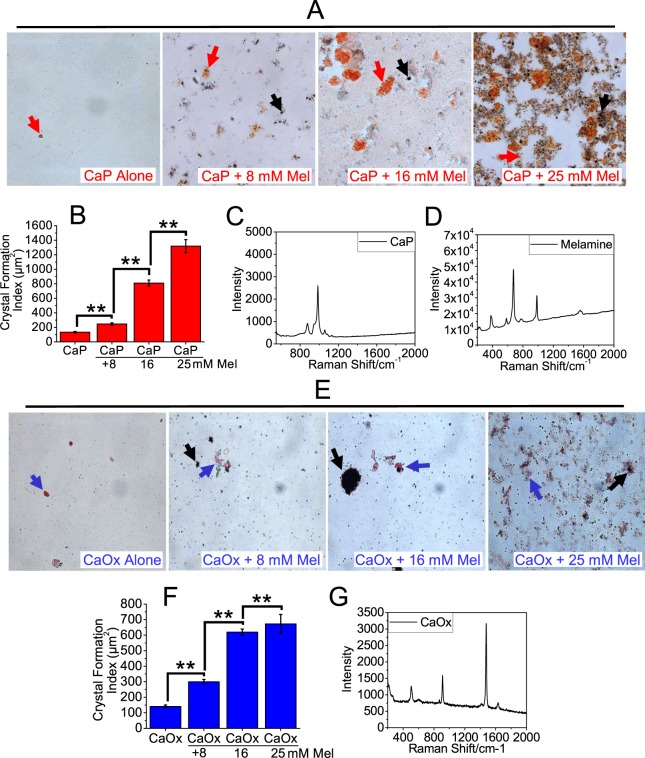
Melamine (Mel) promoted CaP and CaOx crystal formation in non-cellular environment. To observe Mel promoted CaP crystal formations, solutions with CaP alone, CaP with 8 mM of Mel, 16 mM of Mel and 25 mM of Mel, were constituted and stained with alizarin red 4.3 (**A**). Relative crystal abundance was quantified following staining and is represented as a bar diagram. Values are expressed in mean + SEM. *p < 0.05; **p < 0.01 (**B**). Confocal Raman Spectra analysis confirms identity of CaP crystal (**C**) and Mel (**D**). To observe Mel promoted CaOx crystal formations, solutions with CaOx alone, CaOx with 8 mM, 16 mM and 25 mM Mel concentrations, were constituted and stained with alizarin red 6.8 (**E**). Relative crystal abundance was quantified following staining and is represented as a bar diagram (**F**). Confocal Raman Spectra analysis confirms identity of CaOx crystal (**G**). Stained Mel crystals are pointed out by black arrows, stained CaP crystals are pointed out by red arrows, and stained CaOx crystals are pointed out by blue arrows. Images at 20x magnification.

To examine the effect of melamine on the formation of CaP crystals; we first sought to test the effect of adding melamine solution to CaP saturated solution in a non-cellular environment. We added CaP solution alone as control and melamine in a dose dependent manner (8 mM, 16 mM and 25 mM), to CaP solutions (Fig. 2A). The resulting crystals were stained with alizarin red stain (pH 4.3) which stains CaP crystals, and oil red O which stains melamine specifically, and the area of the stained crystals was then quantified by a blinded observer as described and expressed as relative crystal abundance in bar diagram (Fig. 2B). The identity of CaP and melamine crystals was confirmed by Raman spectroscopy and the resulting spectra were obtained (Fig. 2C,D). Our results showed that melamine promoted CaP crystal formation in a concentration dependent manner as defined by the increase in the area of CaP crystals from 8 mM to 25 mM melamine concentration. Significantly, these data provided evidence for the first time that melamine promotes CaP crystal formation, which could have implication in CaP kidney stone formations. In addition to CaP, most of the calcium kidney stones are composed of CaOx. Therefore, we asked whether melamine would have a similar effect on CaOx crystal formation. We added CaOx solution alone as control; and added melamine in a concentration dependent manner (8 mM, 16 mM and 25 mM), to saturated CaOx solution. CaOx and melamine crystals were stained with alizarin red stain (pH 6.8) and oil red O and representative images were obtained (Fig. 2E). Stained crystals were quantified as before and represented as bar diagram (Fig. 2F). To confirm the identity of CaOx crystals, CaOx crystals formed from addition of melamine (16 mM) were analyzed by Raman spectroscopy and the resulting spectra was obtained (Fig. 2G). Our data showed that melamine promoted formation of CaOx crystals in a concentration dependent manner in a non-cellular environment and we confirmed that the crystals formed were CaOx, suggesting that melamine could contribute to the crystallization of both CaP and CaOx.

### Melamine accelerates calcium crystal formation in a time-dependent manner with further increase in the size of the crystals and stabilize the crystal (retention)

**Figure 3:**
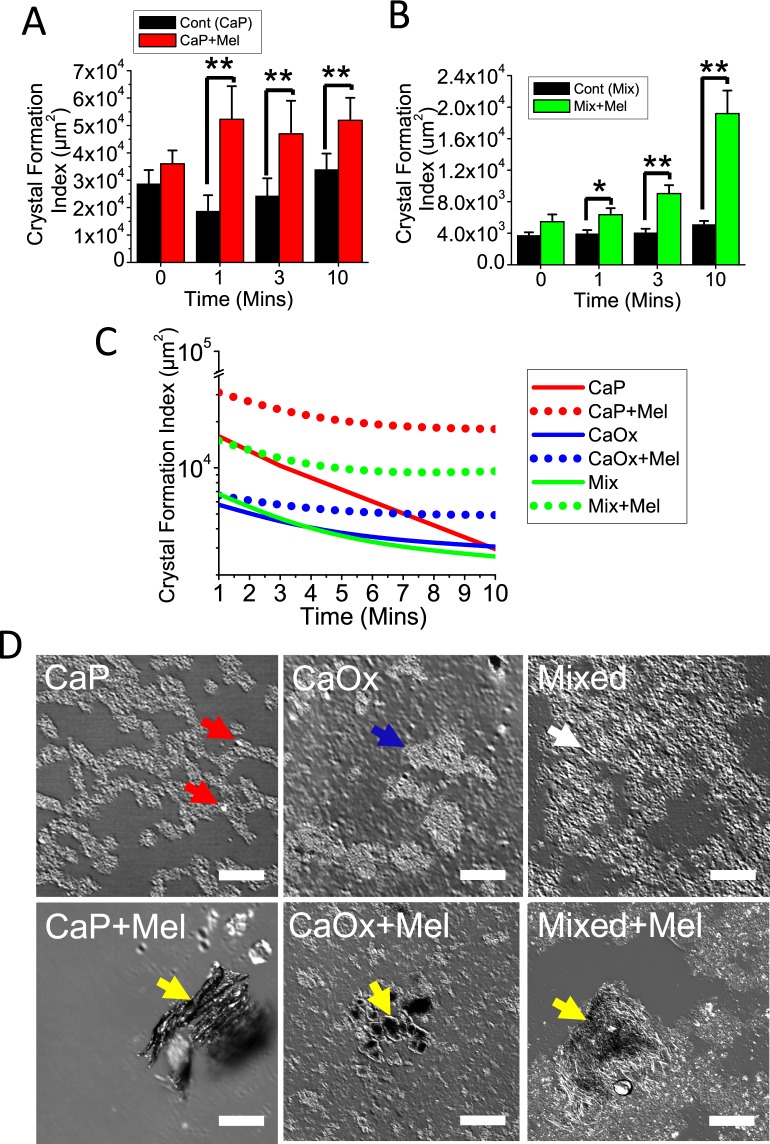
Melamine (Mel) induced CaP, CaOx and mixed crystal growth in a time-dependent manner and stabilizes calcium crystals. Time-dependent effect of Mel on CaP and mixed crystal formation was assessed after addition of Mel (3 mM). Crystals were formed at the appropriate time points (0, 1, 3, and 10 min), and stained with alizarin red 4.3 (**A**) or alizarin red 6.8 (**B**). Crystal formations were imaged and the crystal formation (area) was quantified using ImageJ software and represented with bar diagrams. Values are expressed as mean + SEM. *p < 0.05; **p < 0.01. Effect of Mel on crystal stability was assessed by dissolution experiment. CaP, CaOx and (CaP + CaOx) mixed crystals were synthesized and dissolved in ddH_2_O at RT with or without Mel up to 10 mins. Following dissolution, remaining crystals were collected and stained with alizarin red 4.3 (CaP), or alizarin red 6.8. Stained crystals were imaged and quantified using ImageJ software (**C**). Representative images were obtained with confocal microscope (**D**). Red arrow indicates CaP, blue arrow indicates CaOx, white arrows indicate mixed crystals, and yellow arrows indicate crystals mixed with Mel. Scale bars, 50 µm.

To evaluate the time-dependent effect of melamine on crystal formation, we performed a time-course experiment using saturated CaP, CaOx and mixed solutions, incubated with or without melamine (3 mM) for 0, 1, 3 and 10 mins, and we stained resulting crystals with alizarin red (pH = 4.3 and 6.8). Alizarin red 4.3 stain revealed that average (CaP) crystal size increased over time when solutions were incubated alone, without melamine, with peak crystal size at 10 mins. Significantly, we found that at all time points, melamine induced greater crystal size compared to saturated solutions alone (Fig. 3A). We also assessed crystal size with alizarin red 6.8 stain and found that melamine enhanced (mixed) crystal size over time, with peak crystal size at 10 min (Fig. 3B). Next, we examined the effect of melamine on crystal dissolution. Here, we prepared CaP, CaOx and mixed crystals, and dissolved them in solution with and without melamine (3 mM) for 1, 3 and 10 minutes. Following dissolution, we stained remaining crystals with alizarin red (pH = 4.3 and 6.8) and evaluated crystal size and obtained representative images (Fig. 3C,D). Interestingly, we found that melamine stabilizes the crystal and retarded CaP, CaOx and mixed. Taken together, our results show that while crystal size increased over time when saturated solutions were incubated alone, melamine had the effect of accelerating crystal growth. Further, melamine significantly stabilized crystals and reduced the rate of dissolution^31^. Results presented above suggest two possible explanations: 1. Melamine could have ability to form more CaP/CaOx crystals by augmenting the forward reaction (Supplementary Fig. 2A). 2. Melamine could have the ability to stabilize calcium crystals preventing from dissolution. All of these can exert the growth-promoting effect on calcium crystal formation.

### Melamine enhanced CaP crystal formation in PT cellular environment

**Figure 4:**
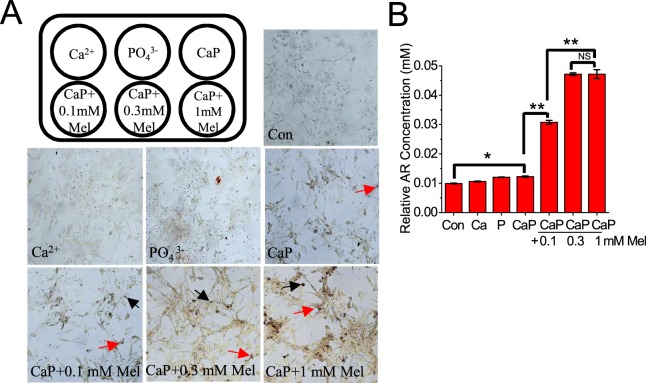
CaP crystal formation by melamine (Mel) in PT cellular environment. Mel enhanced CaP crystal formation in PT cellular environment. 2D cellular environment with primary (mouse) PT cells incubated with a combination of cell culture media (Con), Ca^2+^ alone (Ca^2+^), CaP, and CaP mixed with Mel 0.1 mM, 0.3 mM and 1 mM (**A**). Stained Mel crystals are indicated with black arrows and stained CaP crystals are indicated with red arrows. Images at 20x magnification. CaP crystals formed were stained with alizarin red 6.8 stain and the resulting stain was extracted, quantified and is shown as bar diagram (**B**); values are expressed as mean + SEM. *p < 0.05; **p < 0.01.

Proximal tubular cells (PTC) have been shown to be more susceptible to injury from crystal formation than cells of distal tubular origin^30^. To further our study, we examined the effect of melamine on the formation of CaP crystals in a PTC cellular 2D environment (Supplementary Fig. 2A) as we have described in our previous report^18,20^. Here, we applied Ca^2+^ solution alone, PO_4_^3−^ solution alone and saturated CaP solution alone as controls on the monolayer of HK2 2D cell culture (media alone was used as an internal control). We then added increasing melamine concentrations to saturated CaP solution in the presence of PTCs at increasing melamine concentrations (0.1 mM; 0.3 mM and 1.0 mM) (Fig. 4A). The resulting CaP crystals were stained with alizarin (pH 4.3) and oil red O stain, extracted and quantified as described before, and represented as a bar diagram (Fig. 4B). Interestingly, our data shows that melamine increased the relative abundance of CaP crystals in the presence of PT cells up to 0.3 mM melamine, no significant increase in crystal abundance was observed at 1.0 mM melamine. These data provided further evidence that melamine contributes to the formation of CaP crystals in a cellular environment and could be involved in the formation and growth of kidney stones.

### Melamine promoted CaP, CaOx and mixed crystal formation *in vivo*-like 3D MF device

**Figure 5:**
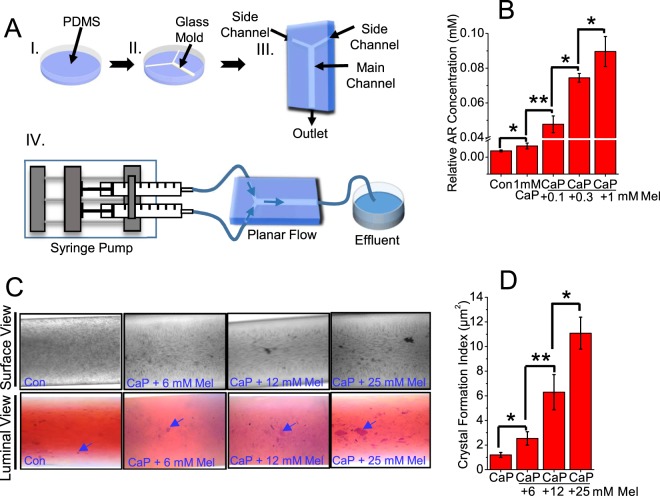
Melamine (Mel) induced CaP crystal formation in *vivo*-like 3D MF device cellular environment. Polydimethylsiloxane (PDMS) MF device channels schematics for simulating an *in vivo* cellular environment. One layer of PDMS was poured into the petri dish (I) and glass pipettes were arranged into desired pattern (II). After another layer of PDMS was poured into the petri dish, the PDMS was cured and the glass template was removed (III). Syringe pump was connected to the MF device for delivery of solutions (IV; **A**). Mel promoted CaP crystal formation in 3D MF device which simulated the cellular environment. To analyze effect of Mel on CaP crystal formations in a 3D cellular environment, HK2 cells were seeded in MF devices for 12–18 hours, and the following day, additions of CaP alone, CaP with Mel concentrations 0.1 mM, 0.3 mM and 1 mM were incubated in the channels and crystal formations were observed and stained with alizarin red 4.3 (**B**). To analyze effect of Mel on CaP crystal formations in a 3D cellular environment, at higher Mel concentrations, HK2 cells were seeded in MF devices 12–18 hours, and the following day, additions of CaP alone, CaP with Mel concentrations (6 mM, 12 mM and 25 mM) were incubated in the channels and crystal formations were observed and stained with alizarin red 4.3 stain (**C**). Stained CaP crystals are pointed to by blue arrows. Stain was extracted and quantified and is represented as bar diagram (**D**). Images at 20x magnification. Values are expressed as mean + SEM. *p < 0.05; **p < 0.01.

To explore the pathological relevance of the effect of melamine on crystal formation we utilized biomimetic MF devices in which CaP and melamine were added to MF channels in a cellular environment and at physiologically relevant concentrations. MF channels were fabricated in a Y-shape to mimic the renal microenvironment as we have previously shown^11^ (Supplementary Fig. 2B). MF channel design is illustrated (Fig. 5A). Here we cultured HK2 cells in the MF devices forming a monolayer, and added CaP alone and CaP with increasing concentrations of melamine (0.1 mM; 0.3 mM and 1 mM). We chose the concentration range to mimic the amount of melamine that may leak from kitchenware into the diet. The CaP crystals were stained with alizarin red stain (pH 4.3) as before, stained crystals were extracted and quantified as shown (Fig. 5B). Our results showed a concentration dependent increase in relative CaP crystal abundance when melamine was added to CaP solution in the MF channel. To further demonstrate CaP crystal formation in MF devices, we increased the amount of melamine added to saturated CaP solutions (6 mM, 12 mM and 25 mM). These concentrations represent accumulated melamine in the renal environment that could contribute to CaP crystal formation. CaP solution was added to MF device and then melamine was added to MF device via another channel at 6 mM, 12 mM and 25 mM concentrations. CaP crystals formed were stained with alizarin red 4.3 stain and representative images were obtained, the surface view and the luminal view of the 3D channels are shown (Fig. 5C). We observed that melamine enhanced CaP crystal formation in a concentration dependent manner but significantly, addition of melamine did not lead to crystal aggregation. Stained CaP crystals were extracted and relative crystal abundance was quantified (Fig. 5D). We observed that melamine enhanced CaP crystal formation in a concentration dependent manner but significantly, addition of melamine did not lead to crystal aggregation.

### Melamine in presence of preformed CaP crystals intensified mixed crystal formation

**Figure 6:**
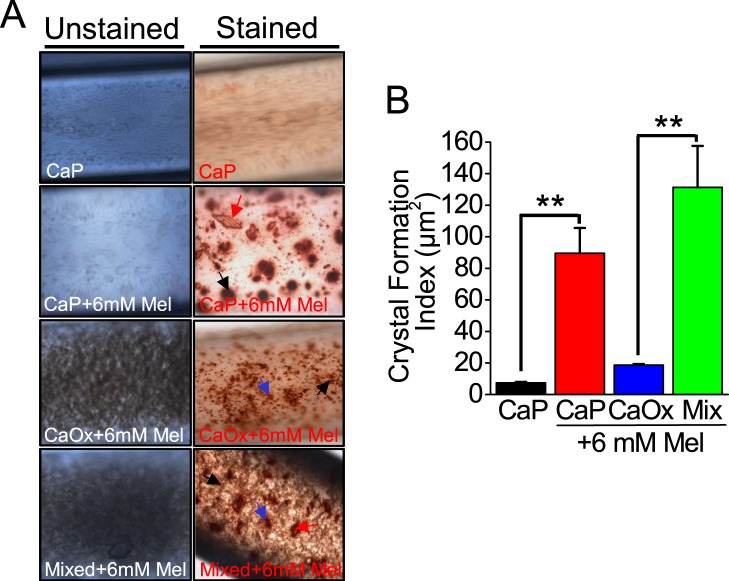
Preformed CaP crystal promoted mixed crystal formation in 3D MF device in cellular environment. To analyze effect of preformed CaP and melamine (Mel) in a 3D cellular environment, HK2 cells were seeded in MF devices 12–18 hours and the following incubation additions of preformed CaP, CaP and 6 mM Mel concentration; CaOx and 6 mM Mel; and CaP, CaOx, and 6 mM Mel were incubated in the channels and crystal formations were observed and stained with alizarin red 4.3 stain (**A**). After collecting microscopy images, crystals were quantified ImageJ software (**B**). Stained Mel crystals are indicated with black arrows, stained CaP crystals are indicated with red arrows, and stained CaOx crystals are indicated with blue arrows. Images at 20x magnification.

We and others have proposed that preformed CaP crystals acts as a nidus for mixed crystal formations^36^, and in clinical condition such mixed crystals presented among the majority of stone formers^37^. Therefore, to produce *in vivo* stone formation scenario, we used preformed CaP crystals to effectively simulate such CaOx crystal formation. Since we found that melamine enhanced CaP crystal formation in MF devices, we also examined whether melamine would have any effect on CaP induced mixed crystal formation. To this end, we added to 3D MF cellular environment- preformed CaP (alone), preformed CaP crystal with 6 mM melamine, saturated CaOx with melamine (6 mM) and a combination of CaP preformed crystal, saturated CaOx and melamine (6 mM), to 3D MF cellular environment, the resulting crystals were stained with alizarin red 6.8 and unstained and stained images were obtained and quantified (Fig. 6A,B). Significantly, our results showed that addition of preformed CaP crystal in the presence of melamine enhanced CaP crystal formation. Interestingly, we found that addition of melamine to preformed CaP crystal induced the formation of larger CaOx and mixed crystals compared to preformed CaP crystals alone. These results thus suggest that preformed crystals exert much greater affinity to induce mixed calcium stone formation compared to the supersaturated Ca^2+^ solution.

### Melamine in presence of preformed CaP crystals induced the stone-forming pathway activation in cellular 3D MF

**Figure 7:**
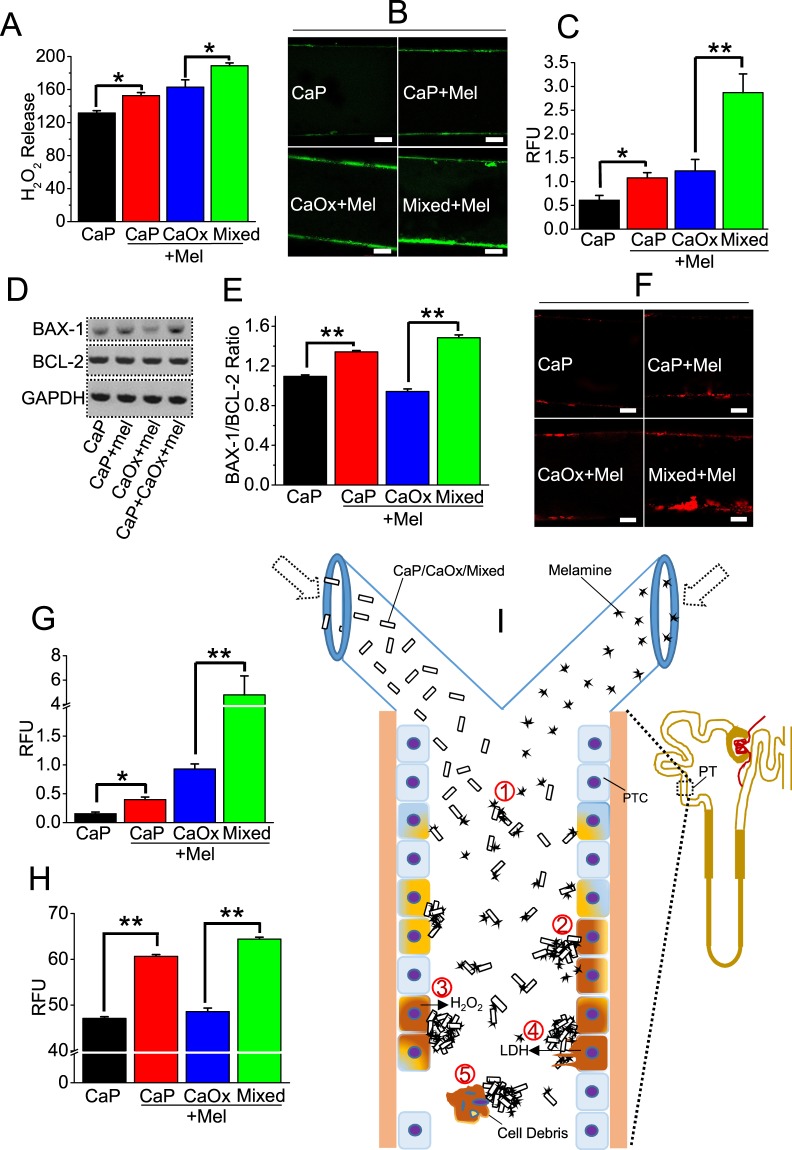
Preformed CaP crystals and melamine (Mel)-induced CaOx crystal formation increases ROS release, LDH release, necrosis and apoptosis. To evaluate the effect of preformed CaP and Mel-induced CaP/CaOx crystal formation on 3D cultured HK2 cells, we measured H_2_O_2_ release (unit: relative fluorescence unit; RFU) representing ROS production (**A**); cells undergoing apoptosis, Alexa Fluor-labeled annexin-V staining (**B**,**C**); RT-PCR blots representing mRNA expression of genes BAX-1 (506 bp) and BCL-2 (147 bp), and GAPDH (336 bp) (**D**). Full RT-PCR blots are provided in the supplementary information. Quantification of BAX/BCL2 ratio (**E**) as apoptosis (normalized to GAPDH as an internal control); PI labeled (**F**,**G**) representing necrosis and cellular damage LDH release (**H**). Schematic diagram showing proposed mechanism **(I)** (1) CaP/CaOx/mixed + Mel crystal interactions. (2) Generates ROS formation within the PT cell. (3) ROS production leads to release of H_2_O_2_. (4) ROS induced cell death via apoptosis and necrosis. (5) Cell debris generated from dead cells helps in further aggregation of crystals. Scale bars in B and F, 50 µm.

To further explore the mechanism of the effect of melamine on calcium crystal formation in PT cellular 3D MF with a greater and intensified effect using preformed CaP crystal, we propose that cellular debris from the injured cells promotes nucleation and aggregation of preformed calcium crystals leading to enhanced crystal formations. We used a HK2 cells, a human PT cell type, because these cells are much more vulnerable to injury/insult and have been proposed as a contributor to calcium stone formation. So, we looked at the effect of melamine in presence of preformed crystals on  ROS production, pro-apoptotic gene activation, cellular apoptosis and necrosis in PT cellular 3D MFs. Here we measured H_2_O_2_ production, as an indicator of ROS production, in supernatant collected from 3D MF in presence of preformed CaP (alone; control), preformed CaP crystal with 6 mM melamine, saturated CaOx with 6 mM melamine, and a combination of CaP preformed crystal, saturated CaOx and 6 mM melamine. Our data showed that the combination of preformed CaP crystals, saturated CaOx and melamine resulted in increased H_2_O_2_ production (Fig. 7A). ROS release and associated ROS cellular damage result in cellular necrosis and apoptosis. To evaluate apoptosis, we stained cells with Alexa Fluor-labeled annexin-V, obtained representative images and quantified fluorescence intensities (Fig. 7B,C). We then measured mRNA expression of BAX and BCL-2 using real time PCR and evaluated BAX/BCL-2 ratio, an indicator of cellular apoptosis (Fig. 7D,E; full gel pictures supplied in Supplementary Fig. 2C). In accordance, we found that the combination of preformed CaP crystals, and melamine resulted in increased BAX/BCL-2 ratio compared to preformed crystal alone, implicating the combination in enhanced cellular apoptosis. We confirmed our findings by evaluating necrosis using PI labeling (Fig. 7F,G) and assessed cellular damage by measuring LDH release (Fig. 7H). Our data showed that the combination of preformed CaP crystals, saturated CaOx and melamine resulted in increased necrosis and apoptosis compared to preformed CaP crystals alone. Taken together, our results show, for the first time, that preformed CaP and melamine promote calcium crystal formations, necrosis and apoptosis under physiological 3D MF constructed with PT cells, suggesting that melamine can create a pathologic condition of mixed calcium crystal formations by modulating the PT cellular microenvironment. We proposed that this mechanism can be used to understand the pathophysiology of calcium kidney stone formation *in vivo* as shown in a schematic diagram in Fig. 7I.

### Melamine promotes growth of CaP and CaOx crystal aggregates

**Figure 8:**
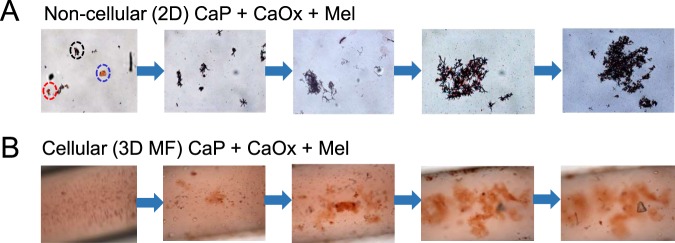
Melamine (Mel) crystals augment growth and aggregation of mixed crystal in both non-cellular (**A**) and cellular environment (**B**) in 3D MF device. Mel was added to mixed crystal and mixed crystal growth and aggregation were observed by staining crystals with alizarin red 4.3 stain. CaP crystal, CaOx crystal, and Mel crystal are indicated by red, blue, and black dashed circles, respectively. Images at 20x magnification.

Crystal aggregation is the process of crystal to crystal binding, resulting in the formation of larger assemblies. Crystal aggregation follows crystal formation and growth, and has been shown to be an important step in stone formation. Since we observed enhanced CaP and CaOx crystal formation and growth in the presence of melamine, we aimed to determine whether melamine enhanced crystal aggregation. Here, we tracked the growth of crystal aggregates in a non-cellular and a cellular environment in the presence of melamine. We observed clearly that melamine enhanced the growth of crystal aggregates over time in a non-cellular environment (Fig. 8A). As well, melamine enhanced the formation of crystal aggregates in a cellular environment (Fig. 8B).

## Discussion

Nephrotoxic effects of melamine have been described to be much worse when combined with cyanuric acid as compared to administered separately. Moreover, the combined administration of melamine and cyanuric acid has the capacity to induce extensive renal crystal formation^38^. Therefore, our first question was whether melamine can pose similar effect on calcium crystal formation. To answer this question, we used a non-cellular environment to avoid the complexity of cellular system and observed that melamine indeed can induce CaP, CaOx and mixed crystal formation almost instantaneously in a concentration dependent manner. We observed spontaneous melamine crystal formations at higher concentration of melamine with the mixtures that contained calcium, phosphate and oxalate ions. The optical morphology and Raman spectra of the crystals were consistent with melamine and CaP/CaOx complex crystals were in good agreement with the peak maximum values obtained for the Raman spectrum of melamine-CaP/CaOx complex crystals that were formed *in vitro*^39,40^. Renal CaP, CaOx and mixed crystal formations are the major cause of kidney stones, the prevalence of which is growing every year. Factors that could be influencing the predispositioninclude persistent exposure to low levels of melamine from sources of our daily use such as plastic plates, cups, and utensils. Such recurrent exposure can predispose to calcium nephrolithiasis due to higher chance of co-crystallization (melamine +CaP/CaOx) and mixed crystal formation.

Our results from the non-cellular environment could have two possible mechanisms: 1. Melamine may have the ability to form more CaP/CaOx crystals by augmenting the forward reaction (Supplementary Fig. 2C). 2. Melamine might also have the ability to stabilize calcium crystals, preventing from dissolution (Fig. 3B). To investigate the first mechanism, we examined the time-dependent crystallization and found that melamine accelerated crystal formation over time, suggesting that the rate of crystallization has been increased. This result could have an implication of mimicking the condition of the descending LOH where tubular fluid becomes more hypertonic (more solutes) and cellular contribution is minimal^41^. Such supersaturated ionic conditions could predispose to form CaP crystals in the  presence of melamine and these preformed crystals can act as a nidus for mixed crystals formation downstream in distal tubule/collecting duct^36^. To address the second possibility, we performed crystal dissolution experiments in presence and absence of melamine and found that melamine had a stabilizing effect on preformed CaP, CaOx, and mixed crystals. Such decrease in the rate of crystal dissolution may have a pathophysiological implication in calcium nephrolithiasis. Dissolution suppression has previously been reported in sparingly soluble phosphates and indicates the creation of metastable states in which the rate of dissolution plateaus^31^. Many factors including the defective calcium/phosphate ions transport in PT, can lead to supersaturation of those ions and generate CaP crystals at the end of PT, which then enters descending limbs LOH, where these crystals could be even more stabilized by melamine. Immediately after, as those crystals proceed through the ascending limb of the LOH, where sodium ions comes out, so the tubular luminal fluid (filtrate) becomes hypotonic, which can have a desolating effect on the preformed crystals as a preventing mechanism. However, such process could be halted by melamine, by decreasing the rate of dissolution of those crystals and can predispose to stone formation downstream in the tubular lumen, provided other conditions are favorable. Interestingly, we found that melamine in fact increased the size of CaP/CaOx crystals, which also brings another possibility that melamine can alter the structure of the CaP/CaOx crystals. However, to fully answer this question, comprehensive biophysical and structural analyses are needed, which could be our future scope of research.

To begin to understand the physiological relevance of our preliminary findings, we assessed whether melamine would have any effect on calcium crystal formation in cellular environment. We utilized HK2 cells, a human PT cell line, because of its established role in  PT ion transporting epithelia and its susceptibility to injury from calcium crystals, both are pertaining to the mechanism of calcium nephrolithiasis. In our cellular approach, we modeled the PT microenvironment, where PT cells actively participate in ion transport and most of the Ca^2+^ and PO_3_^4−^ are reabsorbed in this region of the nephron, preventing supersaturation, and stopping nucleation of CaP crystals. In comparison to our non-cellular approach, we used lower concentrations of melamine to reduce cytotoxic effect in order to better understand the contribution in PT cellular microenvironment and found that melamine upregulates the crystal formations in a concentration dependent manner. We show here the effect of melamine while mimicking the process of *in vivo* CaP deposition within this *ex-vivo* 2D cell culture, which enables us to systematically evaluate the contribution of melamine in CaP and CaOx crystal formation by analyzing the contribution of each microenvironmental cue (e.g., crystal interaction, cellular regulations, and physiochemical interactions) (Supplementary Fig. 2A).

Because of the apparent limitations of 2D cell culture, such as absence of structural and environmental dynamics of an actual kidney PT nephron, we next utilized cellular 3D MF device to provide confirmatory results for the role of melamine in calcium stone formation. In addition to the dynamic microenvironmental conditions (such as fluidic hydrodynamics and physic-chemical interactions) our biomimetic device has been able to recapitulate the nephron microenvironment and enables us to precisely regulate the spatiotemporal composition of fluids, hydrodynamic flow, and chemical stimuli. In our previous report, we described a simple straight channel built in 3D MF device, which was used as a feasability model to demonstrate the calcium phosphate stone formation *in vitro*^11^. Whereas in the present study we used a multi-channel opening of 3D MF device as an upgraded model to understand the effect of multiple chemicals/toxicants etc. on a variety of crystal formations, defining the process *in vivo*-like model. Our upgraded 3D MF device, from a straight channel to a Y-shaped channel, allows us to avoid the mix-up of the test solutions at the time of delivery. Specifically, with two arms used as inlets to introduce multiple chemicals/crystals at the same time within the main device using a laminar flow^42^. The improved device strategy therefore, has the added advantage of reduced shear force, which reduces mechanical stress on embedded cells and better mimics the physiological microenvironment. Recently, another study examined the use of a 3D MF device with a Y-shaped channel to fabricate the formation of CaOx claimed as a collecting duct model^12^, however, failed to sufficiently model the collecting duct, because they did not use cells, a very critical biological component to mimic the absorptive renal epithelia. While our study mimics the human PT, it takes the approach a step further by implementing the biological component through our novel approach of seeding a monolayer of cells into the multi-channel 3D MF, allowing us to simulate the physiological conditions of the PT more accurately, to test the effect of melamine as an environmental toxicant. The cellular environment created in 3D MF using HK2 cells can recapitulate the physiological conditions in the PT of the nephron with a defined condition and can be controlled as needed. Our MF device strategy makes it feasible to study specific role of multiple chemical/pharmacological agents on calcium crystal formation before taking to a preclinical study. Because, examining such effect in animals requires enormous precision and guided application, gauge the doses in such models in administering any exogenous agent and examining their effect(s) on crystal/stone formation. Moreover, rodent models could often be unreliable for such purpose due to dilution effect and the involvement of intertwining factors. Furthermore, in clinical models we can only catch the event at a single time point by biopsy and/or extraction of stone, which is nowhere near to the information we would need to assess the dynamically active calcium biomineralization process.

Since we found that preformed crystals have a pronounced effect on melamine-stimulated mixed crystal formation in cellular 2D and 3D MFs, we performed additional experiments with such conditions to suggest a mechanism for melamine-induced calcium stone formation. We propose that melamine binds to the CaP/CaOx crystals and can cause enhanced ROS production resulting incellular damage and apoptosis. Furthermore, cellular debris resulting from the injured cells can then promote aggregation and growth of calcium crystals leading to enhancement of crystal formations. The PT serves to reabsorb up to two thirds of filtered salt and water^43,44^, filtrate from the PT is carried by the LOH to the renal medulla and back to the renal cortex. In the descending limb, water diffuses out of the luminal fluid into the hyperosmotic medullary interstitium and this serves to elevate the luminal solute concentration^43,44^. We propose that presence of melamine while luminal solute concentration decreases as filtrate transitions via the hairpin and thin ascending limb and through the thick ascending, makes crystals less prone to dissolution, enhances the saturation of the remaining solute- in our case CaP, promotes crystal nucleation and aids crystal agglomeration leading to larger crystal particles. Additionally, preformed CaP crystals are more harmful than saturated solutions and can be more prone to aggregate with melamine and CaOx crystals, which can exacerbate the formation of crystals or stones. Preformed calcium crystals can promote further crystal nucleation and aids crystal agglomeration leading to larger crystal particles. Further studies are required using 3D MF constructed with multiple cell types to evaluate these proposals and their physiological relevance. We have taken a systematic approach to examine the effect of melamine exposure in non-cellular, cellular 2D, and finally by developing a novel MF-based workbench that can simulate the dynamic biological conditions of an *in vivo* renal tubular system (Supplementary Fig. 2B). Further, our combined *in situ* characterization techniques using *in vivo*-like 3D tubular microenvironment generated in MFs will enable us to achieve a greater understanding of the process of CaP and/or CaOx crystal formation at 3D cellular level (Supplementary Fig. 2B).

Our study is novel in its approach to examine the mechanism of mixed crystal formation in *in vivo*-like tubules with continuous renal fluidic flow. Melamine that comes from environmental sources can cause crystal-cell interaction and thus will help in future study to understand the role of drugs/molecules in retention, dissolution, growth of CaP and/or CaOx crystals within the MF-based ex *vivo* renal tubular structure. Moreover, our strategy will help in future plans and execution of studies to prevent and treat calcium stone disease and may also lead us to discover new insights on the abnormal CaP deposition in soft tissues. Such progress will allow for exploration of exciting research fronts related to the understanding of the molecular and pharmacological basis of CaP crystal formation in vascular and other similar cellular microenvironments.

## Supplementary information

Supplementary Files
