# Supplementary material for: Melamine promotes calcium crystal formation in three-dimensional microfluidic device
Source: Sci Rep. 2019 Jan 29;9:875. doi: 10.1038/s41598-018-37191-5 (PMC6351636; doi:10.1038/s41598-018-37191-5)
Supplement: Supplementary file 1 — Supplementary Files [file 41598_2018_37191_MOESM1_ESM.pdf]

Scientific Reports

Supplementary Information

Melamine promotes calcium crystal formation in three-dimensional microfluidic device

Farai Gombedza[a], Sade Evans[a], Samuel Shin[a], Eugenia Awuah Boadi[a], Qian Zhang[b], Zhihong Nie[b], and Bidhan C. Bandyopadhyay[a]*

A

Primer	Sequence
BAX F	5'ATG GAC GGG TCC GGG GAG3'
BAX R	5'ATC CAG CCC AAC AGC CGC3'
BCL-2 F	5'AAG CCG GCG ACG ACT TCT3'
BCL-2 R	5'GGT GCC GGT TCA GGT ACT CA3'
GAPDH F	5'ATC GTG GGG CGC CCC AGG CAC3'
GAPDH R	5'CTC CTT AAT GTC ACG CAC GAT TTC3'

B

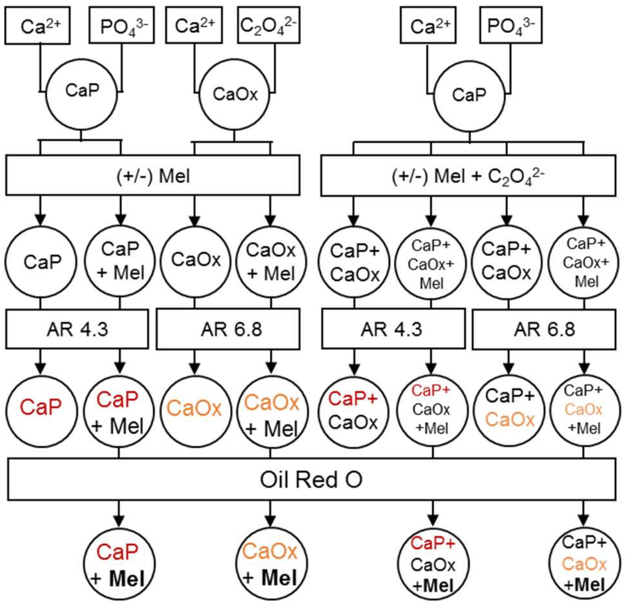

Supplementary Figure 1. Schematic diagram overview of crystal formation and identifications: A. List of primers used for RT-PCR experiments. **B.** Calcium phosphate (CaP), calcium oxalate (CaP), and mixed crystal formation and CaP, CaOx, and melamine (Mel) differential staining with alizarin red (AR) 4.3 or 6.8 and/or Oil Red O staining respectively. CaP

crystals were formed with a mixture of 2.9 mM $CaCl_2$, 6 mM Na_2HPO_4, and 4 mM NaH_2PO_4 while different concentrations of Mel were respectively added to each CaP mixture to reflect concentration variations (A). CaOx crystals were formed with a mixture of 2.4 mM $CaCl_2$ and 4.9 mM $Na_2C_2O_4$ (B); and mixed crystals were formed with a mixture of 2.9 mM $CaCl_2$, 6 mM Na_2HPO_4, 4 mM NaH_2PO_4 , and 4.9 mM $Na_2C_2O_4$ (C). Mel in different volumes were added to different trials of each group respectively to reflect concentration variances. Colored or Bold fonts indicate staining.

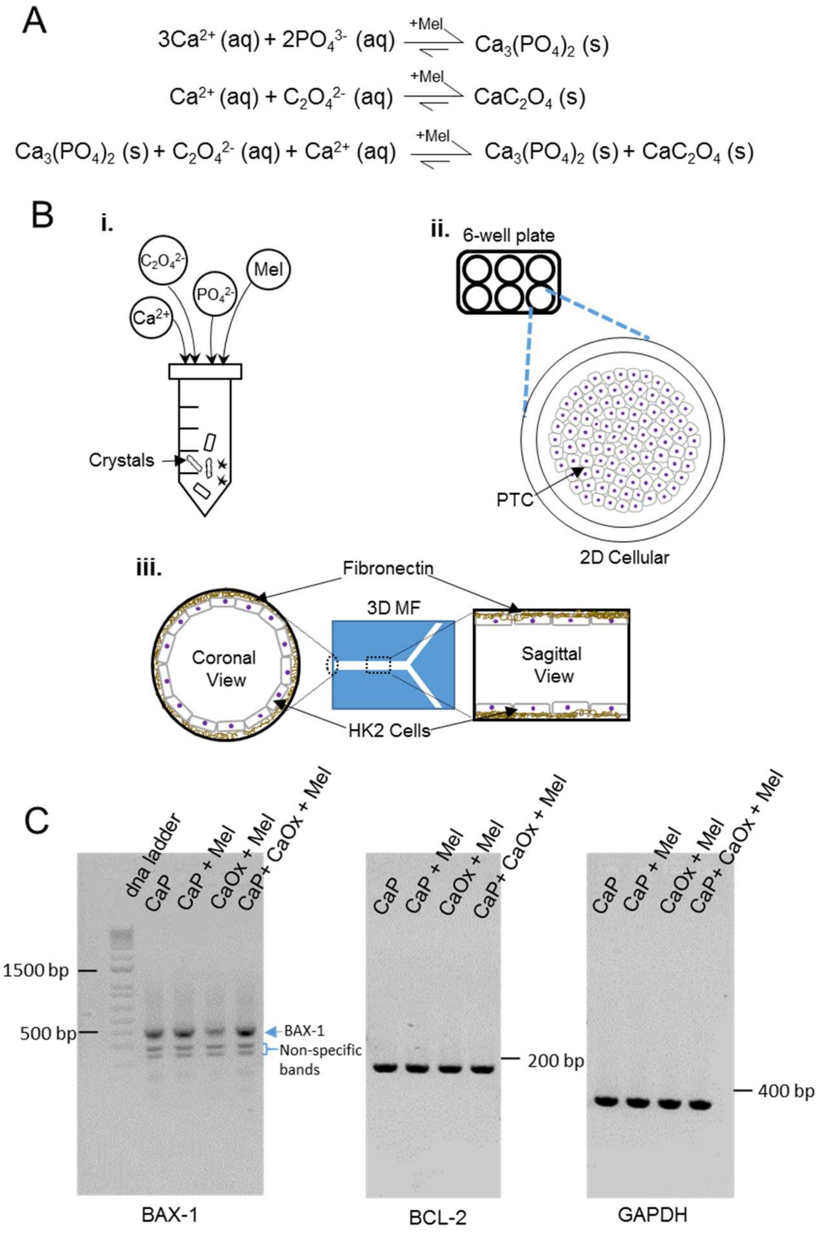

Supplementary Figure 2. Experimental protocols and systems. A. Reaction equation showing crystal formation of calcium phosphate (CaP), calcium oxalate (CaOx), and mixed, where

melamine (Mel) may favor the forward reaction. **B.** Diagrams of preparation for crystal formation in different non-cellular/cellular environments. Mixture of solutions were created to form crystalline structures for CaP, CaOx, and Mel crystals. Graphical schematics of 2-D cellular environment, comprising of a PT cell monolayer and 6-well plate with a zoomed in simple depiction of one well, and 3-D cellular environment within a T-shaped microfluidic device channel with HK2 cells, comprising of zoomed-in simple depictions of i. coronal view and ii. sagittal view of the main channel. **C**. Full RT-PCR blots for experiment performed in 3D MFs detecting apoptotic genes (BAX-1 and BCL-2) and GAPDH as internal control on CaP preformed, CaP+Mel, CaOx+Mel, and CaP+CaOx+Mel treated HK2 cells.
